# The Legacy of #Neurotwitter: A Community at a Crossroads

**DOI:** 10.1212/NE9.0000000000200168

**Published:** 2024-10-31

**Authors:** Zachary London

**Affiliations:** From the Department of Neurology, University of Michigan, Ann Arbor.

In the Spring of 2023, attendees at the American Academy of Neurology (AAN) Annual Meeting had a menu of seemingly redundant sessions from which to choose, including “Tweeting to Take Flight as a Neuro Educator,” “How to Use Social Media as Neurology Teaching Tool,” and “See One, Do One, Teach One: Teaching and Learning With Social Media.”^1^ Overall, the conference program featured 14 live sessions and 5 posters exploring the intersection of career development and the social media platform Twitter. There was even an in-person #Neurotwitter meetup, underscoring the significance of the platform as a tool for education and community building among neurologists. The 2024 AAN Annual Meeting featured only 1 talk and 1 poster about using X (the new name for Twitter) for career development or education.^2^ Anecdotally, the footprint of the once-thriving Neurotwitter community had dramatically diminished in the intervening year. What led to its rise and decline in popularity, and what does the future hold?

The power of Twitter/X as an educational tool is multifaceted. It allows educators at all levels to share knowledge in real time through educational posts, often organized into a series of connected posts known as tweetorials.^3^ The 280-character limit for each post compels an economy of language; those who can concisely express complex topics cater to an audience interested in microlearning, the acquisition of knowledge or skills in small units. Microlearning allows learners to remain up to date, retain knowledge for the long term, and increase engagement in collaborative learning.^4,5^ The mandatory brevity of tweetorials reduces cognitive load.^6^ Users may take advantage of tweetorials asynchronously in a self-directed manner, looking up topics of interest or addressing knowledge gaps. Perhaps more commonly, they may come upon tweetorials randomly when scrolling through recent posts, interweaving educational and noneducational content.

Social media has democratized education, enabling savvy junior scholars, trainees, and other underrepresented voices in medical education to become influential figures on an international stage.

It is in the context of the Neurotwitter boom that Albin et al.^7^ set out to identify the characteristics of a successful neurology tweetorial. They developed a novel metric called the “X Factor” to quantify learner engagement and sharing. They considered author characteristics, content type, visual appeal, and thematic content in 392 neurology-themed tweetorials. They found that the most successful tweetorials had a clearly stated general neurology topic, a creative-first post, an author-made graphic, and unique hashtags. Somewhat surprisingly, longer threads correlated with a higher X Factor, suggesting that depth is valued when presented in a focused, engaging manner.

Twitter was rebranded as X in July 2023, after data collection for this study was completed.^8^ This brought aesthetic changes such as a new logo and new terminology. Tweets were replaced with “posts,” effectively rendering the term “tweetorial” obsolete. The platform's algorithm was adjusted to showcase content from verified users who paid a subscription fee. These paid users' posts were no longer subject to the 280-character limit. This led to reduced visibility of posts made by nonpaying users and diluted the impact of concise, high-quality educational posts. Controversial behavior and polarizing public stances by Elon Musk, the platform's new owner, led to attrition by many users. The rebranded X platform modified its content moderation policy to impose fewer restrictions on post content. Some users left the platform to protest this change, expressing concern that it would lead to the spread of misinformation or harmful content.

Answering the question of whether the Neurotwitter community continues to thrive is not straightforward. It is hard to find trustworthy data about niche social media communities. My observation is that there are still many active users providing high-quality neurology-themed tweetorials and that the platform continues to offer networking and educational opportunities for neurologists and neurologists-to-be. However, some of the more prolific educators from the pre-X era have left or have scaled back their engagement. Although new voices have emerged, they have not reached the prominence or numbers to fill the void left by this attrition.

If Twitter/X is losing its status as the touchstone for microlearning in neurology, will another platform take its place? Competing text or image-based platforms (Instagram, Threads, Facebook, Doximity, Bluesky, Mastodon, and others) have unique advantages and devoted followings, but none of these have emerged as the obvious heir apparent for microlearning content. YouTube and TikTok may offer viable alternatives. TikTok videos typically last under a minute, perfectly catering to microlearning. However, the barriers to creating high-quality video content can feel daunting. Unlike posting on X, where educators can focus solely on their message without concerns for appearance or voice, video platforms require an additional layer of preparation and presentation that may be challenging for many.

We are at a crossroads (or should I say an X-roads?). Perhaps the neurology community on X could return to its former level of engagement and influence. Alternatively, we could see high-profile educators leverage the principles described by Albin et al., such as the importance of a clearly stated topic and creative engagement, to drive a critical mass of medical education enthusiasts to another platform. Hopefully, the spirit of innovation and connection that Twitter/X fostered in its heyday will allow us to find a home and inspire future efforts in neurology education.

## References

[1] American Academy of Neurology 2023 Annual Meeting. Accessed August 19, 2024. aan.com/MSA/Public/Events/Index/45.

[2] American Academy of Neurology 2024 Annual Meeting. Accessed August 19, 2024. aan.com/msa/Public/Events/Index/49.

[3] Albin C, Berkowitz AL. #NeuroTwitter 101: a tweetorial on creating tweetorials. Pract Neurol. 2021;21(6):539-540. doi:10.1136/practneurol-2021-003116 34675121

[4] De Gagne JC, Park HK, Hall K, Woodward A, Yamane S, Kim SS. Microlearning in health professions education: scoping review. JMIR Med Educ. 2019;5(2):e13997. doi:10.2196/13997 31339105 PMC6683654

[5] Thillainadesan J, Le Couteur DG, Haq I, Wilkinson TJ. When I say … microlearning. Med Educ. 2022;56(8):791-792. doi:10.1111/medu.14848 35654438 PMC9542948

[6] Goldstein J, Martindale JM, Albin C, et al. Be in the digital room where it happens, part II: social media for neurology educators. Child Neurol Open. 2023;10:2329048X231169400. doi:10.1177/2329048X231169400 PMC10126786 37114070

[7] Albin CS, Ma T, Pucci GF, et al. Education research: making a tweetorial fly: features of educational social media posts associated with high sharing and engagement. Neurol Educ. 2024;3(4):e200160. doi:10.1212/NE9.0000000000200160. PMC11744482 39834900

[8] Twitter rebrands to X. The Independent. Published July 24, 2023. Accessed August 19, 2024. independent.co.uk/tech/twitter-rebrand-x-logo-elon-musk-b2381006.html.

